# The best tool for the assessment of developmental disorders in children with down syndrome: comparison of standard and specialized growth charts - cross sectional study

**DOI:** 10.3389/fendo.2022.928151

**Published:** 2022-08-05

**Authors:** Marta Hetman, Helena Moreira, Ewa Barg

**Affiliations:** Department of Basic Medical Sciences, Wroclaw Medical University, Wroclaw, Poland

**Keywords:** Down syndrome, growth charts, childhood malnutrition, childhood disability, obesity

## Abstract

Down Syndrome (DS) is a chromosomal abnormality associated with a spectrum of cognitive and physical disabilities. Children with DS are exposed to both lower and excess body weight and follow distinct growth-curve patterns that deviate significantly from those of children without chromosomal defects. Anthropometric parameters are assessed in the pediatric population with the use of growth charts. The study is based on data from 411 children and adults with DS from Poland. Detailed information concerning children and online survey results were also analyzed. Centiles and standard deviation scores (SDS) of obtained anthropometric parameters were aligned with the data using the LMS method. The study aims to identify which type of growth chart (standard vs specialized) is a leading tool for earlier detection of developmental disorders in DS. The results obtained in the two types of growth charts differed. The advantage of the specialized growth charts over the standard ones cannot be unequivocally determined. Only the combination of both tools allows to detect the development disorders early in the broadest possible way.

## Introduction

Down Syndrome (DS, also referred to as trisomy 21) is one of the most common chromosomal abnormalities among live-born neonates and is associated with a spectrum of cognitive and physical disabilities, such as congenital heart disease, hypothyroidism, gastrointestinal disorders, and obstructive sleep apnea (1). The occurrence of DS in 95% of cases is related to meiotic non-disjunction causing trisomy of chromosome 21. The other types of trisomy are Robertsonian translocation and mosaic type (2). DS occurs in every 700-1000 live births (3) and its prevalence estimates between 6.1 to 13.1 per 10 000 people (4). It is predicted that 94.4% of children with DS born in 2000 will survive up to 2020, 90.8% up to 2030, and 76.3% up to 2050 (4). Early identification of developmental disorders can improve the quality of life in the future. To extend lifespan and improve the quality of life, the development of children with DS should be controlled with the use of optimal and appropriate tools.

The most often used parameters to evaluate the child’s growth are anthropometric data such as body height and body weight. Body weight, as a single measure, is not sufficient to assess the nutrition of a given individual, therefore in this study nutritional status is analyzed with Body Mass Index (BMI) - a statistical index used to estimate the body fat content. It is worth remembering that this method is not ideal, but it may be the first step in assessing excess body fat. In the pediatric population, the proper assessment of BMI should be conducted on BMI growth charts.

During the first two years of life, children with DS are characterized by reduced body weight (5, 6), which may result from suction/swallowing disorders associated with muscle hypotonia and dysfunctions in the oral motor system (7). In underweight children the weight for the height it’s a good measurement tool for controlling them. After the second year of life, the occurrence of overweight and obesity in children with DS is more frequent than in the general population (the prevalence of obesity at the level of 30-50%) (8–10), thereby increased BMI is common in DS (11).

Statural growth, as an indicator of development, often represents a child’s health status. The growth retardation of children with DS commences prenatally (12). Morris et al. (6) demonstrated that for gestations up to 38 weeks the median birth weight of newborns with DS is similar to that of babies without DS, however, after 38 weeks their median birth weight rises slower than in unaffected babies. Other researchers also indicate a decreased birth weight in children with DS (13). After birth, the growth velocity is most reduced between 6 months and 3 years of age (14). Short stature is a phenotype of DS and can be influenced by genetic components and other factors, such as comorbidities. Styles et al. (15) compared developmental patterns in terms of body weight, height, and head circumference in children with DS compared to children without DS. Appreciable skewness was noted for body weight, which indicates the difference in the initial weight of children with DS compared to those without DS (15).

Growth charts constitute crucial tools used to assess the growth and nutritional status of children. Currently, various growth charts have been developed and adapted to racial and ethnic backgrounds or a given disease that may interfere with the proper development of a child. The most commonly used DS-specialized growth charts in the US are based on work done in 1988 (14). A great number of countries have constructed DS-specialized charts (5, 16, 17-19). Since these specialized growth charts were developed, concerns have been raised regarding their usefulness. Children with DS follow distinct growth-curve patterns that deviate significantly from those of children without chromosomal defects, therefore the use of specialized growth charts appears to be a superior method in development evaluation. The study aims to identify which type of growth chart (standard vs. specialized) is a leading tool for the earlier detection of developmental disorders in DS.

## Material and methods

### Design and participants

A cross-sectional study design was based on data from 411 people with DS: 386 (94%) children and 25 (6%) adults; 188 (46%) girls and 223 (54%) boys, aged 0.17 months – 36.72 years (median: 4.85) from Poland recruited from general pediatric practices and parents’ interest groups. Inclusion criteria were patients with a diagnosis of DS. There were no exclusion criteria. The study was conducted in the years 2020-2021 in Wroclaw (Poland) as a part of the doctoral dissertation carried out at the Wroclaw Medical University. The ethical approval on the research protocol and consent form was obtained from the Bioethics Committee, Wroclaw Medical University (approval number KB 674/2020). The study was carried out in accordance with the Declaration of Helsinki. Administrative approvals were obtained from each institute to access the participants’ data. Written informed consent was obtained from the parents of the participants prior to data collection and anthropometric measurements.

### Data collection

**Figure 1:**
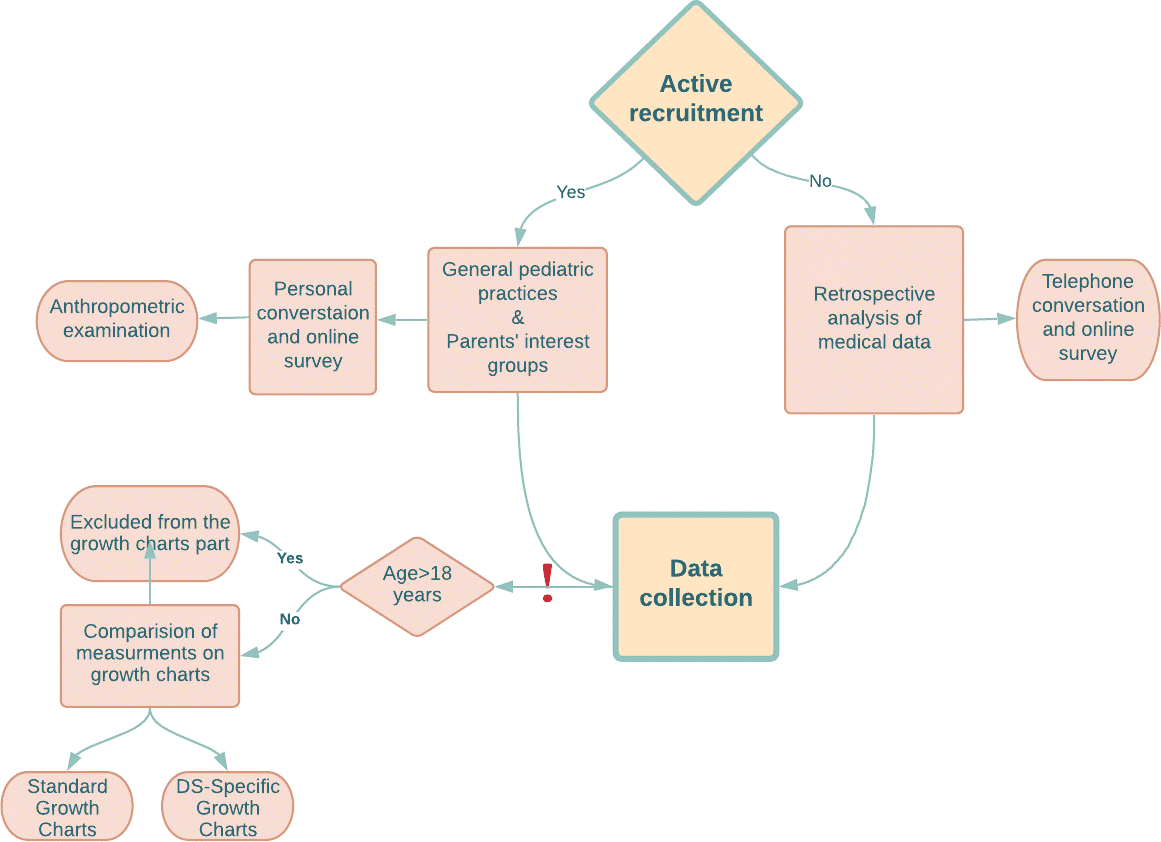
The design of the study (Data Collection).

The data were derived using two approaches between January 2020 - June 2021: by retrospectively examining medical records (20%) available at health clinics (additionally an online/telephone interview with parents or guardians was conducted to confirm the data and by obtaining the consent of data usage) and by actively recruiting participants (80%). Active recruitment and examination of retrospective medical records were conducted among children from all over Poland. All parents were invited to an online survey as an additional part of the study. After written informed consent was obtained, actively recruited children underwent an anthropometric examination in a pediatric clinic in Wroclaw (Poland) with collecting the anthropometric parameters such as body height and weight. For telephone calls, written consent was obtained by sending the consent form online. Then, the parent was asked to return the signed consent by post (original document) or online (scan of the document). Body Mass Index (BMI) was calculated using the formula: weight/height^2^ (kg/m^2^). Trained personnel (consisted of 3 people: two doctors (including authors) and a nurse) obtained measurements following standardized techniques (20), discussed prior to the research initiation. The design of the study (Collection Data part) is represented in **Figure 1**.

#### Specific data and online questionnaire

Specific data were collected from 200-300 participants depending on the parameter. Detailed information concerning parents of children with DS (**Table 1**), the perinatal period (**Table 2**), comorbidities (**Table 3**), and L-thyroxine treatment were obtained. In addition, an online 4-questions questionnaire concerning the topic of growth charts and their usage in medical offices was conducted (**Table 4**). Two hundred eighty-one parents (including those taking part in the main part of the study) answered the survey.

**Table 1:** Parents’ basic characteristics.

		Mother	Father
Age during pregnancy [years]	Average	32.41 ± 5.91	33.92 ± 3.43
	Median	32	33
	Min.	17	20
	Max.	54	58
Body weight [kg]	Average	68.40 ± 12.87	87.27 ± 13.75
	Median	65	86
	Min.	43	57
	Max.	110	130
Body height [cm]	Average	165.64 ± 5.73	179.59 ± 6.91
	Median	165	180
	Min.	147	159
	Max.	178	198
BMI [kg/m^2^]	Average	24.89 ± 4.5	27.02 ± 3.77
	Median	24.17	26.87
	Min.	17.92	18.93
	Max.	39.40	37.55

**Table 2:** Characteristics of postpartum parameters in the group of breastfed-children (85 children; 18 babies were born ≤36hbd).

	Min.	Max.	Median	Average
Birth weight [g]	1490	3970	2800	2840 ± 600
Birth length [cm]	41	59	51	51.29 ± 0.03
Week of pregnancy	31	42	38	37.55 ± 0.00
Apgar score 1’	0	10	9	8.25 ± 0.00

**Table 3:** The prevalence of selected common comorbidities in children with DS reported by parents.

Comorbidities	N	[%]
Hypothyroidism	142	52.98
Vision defects	112	41.79
Cardiac defects	64	23.88
Hearing problems	59	22.01
Immunodeficiency	34	12.69
Others	25	9.33
Lipid disorders	19	7.08
Autoimmune diseases	18	6.71
Malignancy	4	1.49
Hyperthyroidism	3	1.11
Hypertension	2	0.74

*N- the amount of participants (DS) with chosen comorbidity.

**Table 4:** Internet survey (questions with answers).

No.	Question	Answers	Results [%]
1	Have you ever heard about specialized growth charts for children with DS?	Yes	73.3
		No	26.7
2	Which type of charts is more often used by clinicians?	Standard	81.8
		Specific	13.2
3	Have any of the clinicians used specialized charts at least once?	Yes	28.2
		No	71.8
4	Are specialized growth charts important for you as a parent?	Yes	93.2
		No	6.8

The ranges for assessing the parents’ BMI: underweight [15.0, 18.5); healthy weight [18.5, 25.0); overweight [25.0, 30.0), and obesity ≥30The ranges for assessing the birth weight: high birth weight - greater than 4200g; normal weight - 2500g–4200g; low birth weight - less than 2500g; very low birth weight - less than 1500g; and extremely low birth weight less than 1000g.

### Anthropometric measurements

Body weight: body weight (kg) was measured using the same electronic digital scales model (OMRON BF-515) with light clothes and barefoot for older children and without clothing or diapers for infants and toddlers (to the nearest 0.1kg for children >3 years and 0.05kg for children <3 years). Body height: length (to nearest 0.1 cm) was measured on an infant length board (SECA 234) for infants and toddlers unable to stand unsupported (in the supine position). For all others, height (to nearest 0.1 cm) was measured with a stadiometer (SECA 264). The trained personnel controlled the correct body posture of the child during the measurement: straight back, both feet on the ground, back of the body pressed against the wall. The same devices were used for all measurements, without changing the conditions. Birthdate information were extracted from the family or children’s questionnaire. Body weight, body height, and BMI were expressed in the standard deviation score (SDS) value using the LMSgrowth Calculator (21)- a Microsoft Excel add-in. Centiles and SDS were fitted into the data using the LMS method (22). The LMS method summarizes the changing distribution of weight, height, head circumference, and BMI according to age by three curves representing the median (M), coefficient of variation (S), and skewness (L), the latter expressed as a Box-Cox power. The method assumes that the data in each age group can be rendered normally and distributed by applying a suitable power transformation (23). SDS indicates how many standard deviations an observation is above or below the mean independently of age and sex, which is a useful way of putting data from different sources onto the same scale (Equation (1)). With the use of this statistics tool, it is possible to analyze the variability of the observed parameter over a certain period in a group of patients, especially those of developmental age.


(1) $$x\;SDS=\frac{x\;value-x\;value\;for\;50th\;centile}{\frac{1}{2}x\left(x\;value\;for\;50th\;centile-x\;value\;for\;3rd\;centile\right)}$$

(1) x−height/weight/BMI

### Growth charts and data analysis

To standardize the data, the British (24) growth charts included in the LMSgrowth Calculator were used for the calculations and taken as reference for the population of children without DS (population growth charts). DS-specific growth charts were used as the reference point for the population of children with DS (15). Three ranges were assumed (in percentile (PC)): <3rd, 3rd-97th, and> 97th, where 3rd-97th means a wide range of the norm (**Table 6**). However, it should be remembered that values >90th PC should be considered as overweight and that further calculations are related to obesity (>97th PC). Data from people over the age of eighteen (6%) were not considered in the comparison of growth charts. However, their parents were included in the online questionnaire part, mainly referring to their earlier experiences.

### Statistical analysis

The data were processed using Statistica v. 13.3. The data were checked for normality using the Shapiro-Wilk test. Non-parametric statistical tests were applied. The Mann-Whitney U test was used for non-parametric data. Spearman’s rank correlation (r) was performed to investigate the specific data (such as perinatal period, parents’ physical status, L-thyroxine treatment, all affecting the current body height, body weight, and BMI of the child). The chi-squared test was used for data distribution. Descriptive statistics are presented as median/mean ± SD/percentages. P-values <0.05 were considered significant developmental disorders in DS.

## Results

The study is based on data from 411 people with DS. Two hundred and fifty-five people from the study group have simple meiotic non-disjunction trisomy of chromosome 21; 12 are mosaic type; 10 have Robertsonian translocation. In the remaining cases of the questions about the type of mutation, the parents did not provide an answer, did not know the answer to the question, or never tested the child for a given mutation. The mean birth weight was 2898.02 ± 513g (median 2800 g); average birth length 0.52m ± 0.04m (median 0,51). The average age of delivery (weeks) was 37.7 ± 2.17 weeks.

### Parents data

**Table 1** presents the parents’ basic characteristics. Fathers: One hundred thirty-six fathers (69%) have a BMI ≥25 (overweight) of which 31% corresponding to a BMI ≥30 (obesity). The average BMI value is27.02 ± 3.77. Mothers: eighty-four mothers (42%) have a BMI ≥25 (overweight) of which 26% corresponding to a BMI ≥30 (obesity). The average BMI value is 24.89 ± 4.58. There is no correlation between the current weight of the child and the parents’ weight.

### Child’s birth weight

Data on birth weight were collected from 266 children. Fifty-nine babies were born ≤36hbb and were treated as premature babies. Among preterm babies, the mean birth weight was 2872.96 ± 484g (median 2800.00g; min.1490g; max. 3970). Among full-term babies (≥39hbd), the mean birth weight was 2967.97 ± 444g (median 2800g; minimum 2004g; maximum 4500g). Taking into account the entire group of 266 children, the mean birth weight was 2898.02 ± 513g (median 2800 g; min. 1490 g; max. 4500g). A positive correlation (low correlation) (rs = 0.152555) is found between the baby’s birth weight and their current body weight.

### Breastfeeding

Out of 109 children whose parents answered the question about breastfeeding, 85 (78%; girls: 36; 18 babies were born ≤ 36hbd) were breastfed. Max. duration of breastfeeding: 40 months; min. 0.5 months (median 9 months; average 11.06 ± 0.52months). Among those who were breastfed, 39 pregnancies were completed by natural childbirth. There were complications during childbirth in 15 cases. The median duration of pregnancy was 38 weeks (average 37.55 ± 0.00). The most common reasons for not breastfeeding were: lack of suckling reflex in the child and/or lack of lactation in the mother. There was no correlation between breastfeeding and body weight, body height, and BMI of the child. The median Apgar score among children fed breast milk after birth was 9 (average 8.25 ± 0.00). **Table 3** presents basic characteristics of postnatal parameters in the group that was breastfed.

### L-thyroxine therapy

There were 265 responses related to L-thyroxine therapy (L-thyroxine was taken by 169 children, 64%). There is no correlation between L-thyroxine intake and body weight or BMI. However, a statistically significant difference was identified for the body height growth charts readings. Smaller spread of values concerned children taking L-thyroxine. This means that when comparing the group of children taking the L-thyroxine and those not taking L-thyroxine, the children taking the medicine were within the wide normal range (3rd PC- 97th PC) more often.

### Comorbidities

The data relating to selected comorbidities (**Table 3**) were collected from 198 participants. The three most common comorbidities in the study group are hypothyroidism affecting 52.98%; vision defects affecting 41.79%; and cardiac defects affecting 23.88%.

### Growth charts

**Figure 2:**
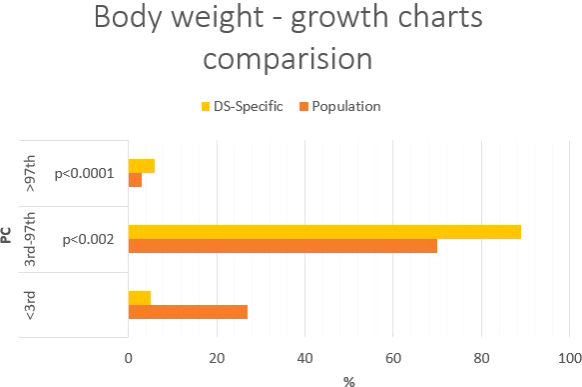
Percentage comparison of the number of individuals qualified for the given body weight categories on the standard growth charts and DS-specialized growth charts.

**Figure 3:**
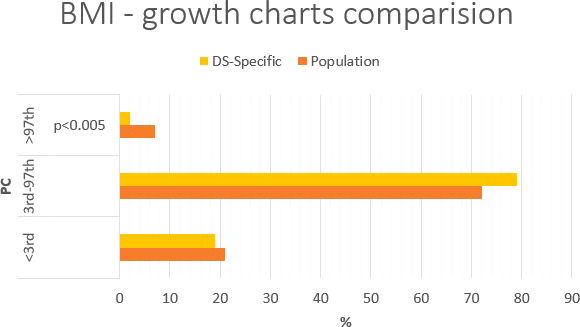
Percentage comparison of the number of individuals qualified for the given body height categories on the standard growth charts and DS-specialized growth charts.

**Figure 4:**
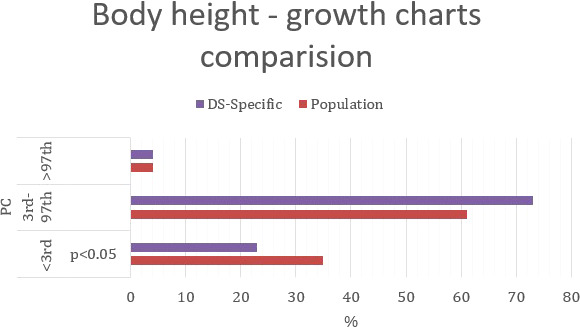
Percentage comparison of the number of individuals qualified for the given BMI categories on the standard growth charts and DS-specialized growth charts.

**Table 5** shows percentiles and corresponding values of the SDS and their interpretation in relation to anthropometric parameters. A graphic representation of **Table 6** is shown in **Figures 2**–**4**. Comparing the results obtained on two types of growth charts (standard vs. specialized): body weight - results outside the norm 30% vs. 11%; body height - 39% vs. 27%; and BMI - 28% vs. 21%. More results beyond the norm (under 3rd PC and above 97th PC) were obtained using standard growth charts.

**Table 5:** Percentiles and corresponding values of the standard deviation score and their interpretation in relation to anthropometric parameters.

Percentile (PC)	Standard deviation score (SDS)	Body weight	Body height	BMI
<3^rd^	<-1.88	Underweight	Growth deficiency	Underweight
3^rd^-10^th^	≥-1.88,<-1.66	Normal	Normal	Normal
10^th^-90^th^	≥-1.66, ≤1.66	Normal	Normal	Normal
90^th^-97^th^	>1.66, ≤1.88	Overweight	Normal	Overweight
>97^th^	>1.88	Obesity	High growth	Obesity

**Table 6:** Distribution of data (body height, body weight, BMI) in both types of growth charts- standard and specialized growth charts.

		Body weight		Body height		BMI	
PC	Classification	StandardN (%)	DSN (%)	StandardN (%)	DSN (%)	StandardN (%)	DSN (%)
<3^rd^	Under	108 (27)	23 (5)	141 (35)	90 (23)	83 (21)	76 (19)
3^rd^-97^th^	Norm	280 (70)	350 (89)	246 (61)	288 (73)	290 (72)	306 (79)
>97^th^	Over	13 (3)	19 (6)	14 (4)	14 (4)	28 (7)	7 (2)

*PC- percentile, DS- Down Syndrome specialized growth charts, N- amount of individuals.

#### Body weight

Using standard growth charts to assess body weight in a child with DS instead of the specific charts, 19 percentage points (p.p.) fewer children were considered in the range of normal body weight (70% vs. 89%, p<0.02), 21 p.p. more children had body weight deficiency (27% vs. 6%, p<0.0001); 2 p.p. fewer children had excess body weight (3% vs. 5%) (**Figure 2**). The statistically significant difference was observed in groups with weight deficiency and normal body weight.

#### Body height

Using standard growth charts to assess body height in a child with DS instead of the specialized growth charts, 12 p.p. fewer (61% vs. 73%) children can be included in the normal range; 12 p.p. more (35% vs. 23%; p<0.005) children is above 97th PC (**Figure 3**). There is no difference in groups <3rd PC (4% vs. 4%) The statistically significant difference was observed in group with body height >97th PC.

#### Body mass index

Using standard growth charts to assess BMI in a child with DS instead of the specialized ones, 7 p.p. fewer children were considered in the range of normal BMI (72% vs. 79%), 5 p.p. more children had BMI <3rd PC (7% vs. 2%); and 2 p.p. more children had BMI >97th PC (21% vs. 79% p<0.005) (**Figure 4**). The statistically significant difference was observed only in group with BMI>97th PC.

### Online survey

Two hundred and eighty-one people (parents of DS people) answered the questions from the Internet survey. The survey deals with the topic of growth charts and their application in clinical practice by doctors. The condition for completing the survey was answering all the questions. The results of the online survey are shown in **Table 4**.

## Discussion

In this study we compared the assessment of children and adolescents’ development in terms of body weight, body height, and BMI, using growth charts for the standard population and the sub-population of people with DS. The main objective was to identify which type of growth chart is the best tool for earlier detection of developmental disorders in DS. The results obtained on the two types of growth charts differed. Due to numerous comorbidities, disease phenotype, and social conditions, people with DS can be considered as a vulnerable population that requires systematic monitoring of their health status. Advances in medical care and increased access to knowledge have improved the health and well-being of individuals with DS. Currently, the illusion created in society is that the number of people with DS is decreasing. However, children with DS, one of the most common chromosomal abnormalities, will continue to be born, and with the current medical knowledge their lives may be longer, better and healthier. Monitoring of the child’s health by doctors and parents should be performed with the use of appropriate assessment tools, of which the simplest and most common are growth charts. The challenge is to choose the kind of growth charts for the assessment of a given parameter so that the obtained results have a real impact on clinical decisions. The original hypothesis assumed that DS-specialized growth charts are chief tools in the comprehensive assessment (body weight, body height, BMI) of the developmental disorders in a child with DS. The obtained results, combined with clinical knowledge and experience, appear to contradict this hypothesis.

### Body weight and BMI

Monitoring the child’s development from the earliest stages of life with the use of optimal tools gives a chance to improve their quality of life in the future. Early health intervention can meaningfully affect adulthood. The problem of weight disorders among people with DS is very complex and challenging, concerning mainly the rapid transformation between undernutrition in the first period of life and excessive weight gain in later years. Therefore, depending on the age, this population is exposed to both deficiency and excess body weight and all the associated health consequences. As mentioned earlier, children with DS are characterized by a lower birth weight than children without chromosomal abnormalities (5, 6). However, in adolescence and adulthood, due to numerous comorbidities and the characteristics of the syndrome itself, people with DS are exposed to excessive body weight. Systematic nutritional evaluations since the day the baby is born throughout later years of life is essential. A higher obesity rate compared to the general population is observed among adolescents and adults with DS (25), therefore prevention and early treatment are principal aspects. DS has traditionally been considered as an “atheromafree” condition (26), however, the recent studies appear to contradict this thesis (27, 28). BMI, as based on body height and body weight, is a superior indicator of body nutrition. Both BMI and body weight are assessed using growth charts to detect body weight disorders. Hatch-Stein et al. (17) observed that for individuals with DS, the 85th percentile on standard growth charts is a better indicator of excess adiposity than the 85th percentile on the DS-specific BMI growth charts and claimed that standard charts should be the preferred method for early identification of obesity in children with DS. The results obtained in our study appear to confirm this. The percentage of out-of-normal results was analyzed, yielding a higher results percentage for body weight and BMI when using standard growth charts instead of the specialized ones. The use of specialized growth charts can deceptively reassure parents and lulls doctors into a false sense of a child’s security. Since DS from adolescence is predisposed to excess body weight and has an increased risk of cardiovascular disease, the assessment of their BMI should be more rigorous, hence we recommend using standard growth charts. The percentage of children with DS with excess body weight is increased by genetic predisposition, low physical activity, and a high-calorie diet. Poor knowledge of healthy foods has been described in children and adolescents with DS (29). Increasing physical activity should be carried out wisely. Due to the cardiological burden in the group of people with DS, it may be necessary to assess the body’s efficiency and consult a cardiologist before increasing physical activity. Introducing a balanced diet and regular meals should not be neglected. All the above-mentioned activities should be carried out with comprehensive care of i.e. the physician, nutritionists, psychologist, or trainer. A tremendous role in the whole process is played by parents and guardians. The whole family should be characterized by proper nutrition and activity patterns. Our study indicates that parents also face the problem of being overweight and obesity, which is particularly illustrated by the high BMI of the fathers (however, there is no correlation between the current weight of the child and the parents’ weight.). Fortunately, the awareness of parents who are trying to limit flour products and products with high sugar and saturated fat content in their children’s diet is growing. Nevertheless, this challenging task is becoming very difficult to implement as the child grows older. However, it is not only the excess body weight that is a problem. In the study group, 1/5 of children were born with low birth weight. Low body weight in the first stage of a child’s development may result from both maternal and child factors. On the side of maternal factors, there are, among others, problems with lactation, a lack of willingness to breastfeed, stress, and being overwhelmed by a new life situation related to childbirth (30–32). Breastfeeding in DS children is possible and preferred. A chance for its success can be obtained with the appropriate support of the family and competent health professionals. Frequent feeding problems in DS are the lack or very weak suckling reflex, prematurity, and defects in the digestive tract (33). Heart defects, which cause great and quick fatigue in newborns, contribute to the weak sucking reflex. Similar problems were confirmed in specific data collected in our study. Feeding difficulties, slow weight gain, and its deficiency may result in a slow and significantly impeded psychomotor development of a child. Introducing new products to a child’s diet should take into account not only the type of product but also its texture (e.g., small pieces, mousses). Reduced feeding abilities with the increased risk of dysphagia and aspiration are predominant in the first years of life (32). If a child with DS is found to undergo weight loss and/or slow weight gain referring the child for a video-fluoroscopic swallow assessment and the diagnosis of contributing diseases (e.g., heart defects, celiac disease, gastrointestinal defects (Hirschsprung’s disease, duodenal atresia, and others)) should be considered (32). Very important is the detection of disorders associated with both in deficiency and excess body weight and the approach of steps designed to fix these disorders.

### Body height

People with DS are characterized by different patterns of growth compared to children without DS. The greatest impairment can be observed between 6 months and 3 years of age and in the puberty period, when they reach their final height (at age 15-16 years) (34). Furthermore, a shorter and earlier puberty spike related to the earlier achievement of the target height (girls: average of 9.5 years old, boys: average of 11 years old) is observed (35, 36). The assessment of body height using standard growth charts may be unfavorable as short stature is the phenotype of DS. However, the final growth of children with DS depends both on the characteristics of trisomy 21 and on the genetic potential transmitted by parents. The administration of growth hormone (GH) therapy in children with DS is associated with numerous controversies. Palloti et al. (37) observed in attempts of 3-year GH treatment an average improvement in the final height (boys by 5.16 cm; girls by 7.35cm). On the contrary, the other study shows that early treatment with GH does not affect the improvement of final height, but has a positive effect on psychomotor development and increases head circumference (38). In GH therapy a very problematic issue concerns the high risk of cancer, especially in the presence of the Philadelphia chromosome. Administration of GH could increase this risk of proliferative processes, consequently, the legitimacy of its administration should be considered. DS-specific growth charts used to assess a child’s body heigh may provide valuable data to parents resulting in perceiving their child within the normal range. This can avoid unnecessary deliberations on the supply of growth hormone, the action of which, as presented above, may also have negative consequences. Additionally, there are risks of diseases that may result in delaying the rate of growth and achieving final growth, such as celiac disease or hypothyroidism. DS-specific charts were created based on data from people with Down syndrome, the presence of child measurements below the lower limit of normal is a signal for medical intervention, hence we recommend using population growth charts to assess the body height in DS.

### L-thyroxine therapy

Many reports suggest that L-thyroxine therapy in the first years of life (also in children without diagnosed hypothyroidism) may result in better psychomotor development, support the child’s physical therapy, and reduce thyroid immunization (39, 40). Thyroid diseases are one of the most common comorbidities among the population of people with DS (41). Most people at various stages of their lives are at risk of developing hypothyroidism. Among our study group, hypothyroidism was the most common accompanying disease in DS. In the study group, children taking L-thyroxine were within the normal range more often. On this basis, it can be concluded that the supply of L-thyroxine may support the proper growth of children with DS (40).

### Comorbidities

Children with DS suffer from many comorbidities that may have nutritional implications and consequences. At the same time, thyroid disease is one of the most common accompanying diseases in DS. Obesity, as a civilization disease, very frequently affects people with DS. Obesity is also known to be associated with type 2 diabetes, cardiovascular disease, metabolic syndrome and some types of neoplastic processes. Complications of obesity and related diseases can cause and intensify neurodegenerative processes (42).

### Parental outcomes

It is well known that the older age of the mother is associated with Down’s syndrome in children (43). According to the data (2011–2015), the average age at birth of the first child was 25.5 years for men and 23.1 years for women (44). In the study population the average age of mothers and fathers was: 32.41 ± 5.91 and 33.92 ± 3.43 years (**Table 1**). It is worth remembering that, paradoxically, taking into account the entire population, more children with DS are born to young mothers as they are of reproductive age. This can of course change over time as more and more women choose to have offspring later in life.

### Online survey

The results of the online survey appear to be disturbing. So far, no growth charts adapted to the population of individuals with DS have been officially developed in Poland. Nevertheless, access to many other DS-specialized growth charts, including those developed by the CDC (5), is quick, simple and common. Despite that, as the survey results indicate, specific growth charts are not used in everyday medical practice in Poland although parents are aware of their existence. There can be many reasons for that state. First, many physicians are unaware of the existence of specialized growth charts. Secondly, in everyday practice, it is easier and more efficient to use standard growth charts. What’s more, standard growth charts are available in every child’s health booklet (parents should have it with them at every medical visit), which makes it effortless and faster to apply the child’s data on the charts. Additionally, many doctors do not believe that it is necessary to use specialized charts to assess the development of children with DS. The usage of DS-specialized growth charts has certain consequences mainly regarding the previously mentioned issue of BMI assessment and the apparent dormancy of the parent’s vigilance, while activities related to reducing the child’s weight should already be taken. The optimal solution seems to be the assessment of a child on both types of growth charts to fully control their development at every stage. Fortunately, many parents are printing specialized growth charts and having them with them or pasting them into a child’s health book. However, the results obtained only on specialized growth charts (body weight, BMI in some cases) may cause the parent to perceive the body weight as healthy even though it may require early intervention. Nevertheless, DS-specialized growth charts should be implemented into pediatric departments as an important and additional tool to properly assess the development of children and adolescents with DS. When extending the scope of research on this topic it would be worth expanding the research group to include people from the medical community.

### Advantages and limitations of the current study

This study should be interpreted in light of its limitations. First, the data we obtained from medical records (20%) could be the results of measurements performed without the use of standardized techniques, and this could lead to measurement errors. Part of the study population came from a pediatric endocrinological health clinic, which means that they are treated for endocrine reasons. At the same time, most children with Down’s syndrome are burdened with comorbidities, so it would be difficult to single out a group for example without heart defects and thyroid problems. A sample of children attending a medical office was used, not a random sample from the target population. Our research, in order to be more valuable, could also be expended by measurement of the head circumference. As the standard growth charts, the 1990 British growth charts were used. This choice was dictated by the high detail of the mentioned growth charts and their coherence with the LMSgrowth Microsoft Excel add-in used. This study also has some strengths. First, the sample size is large. Second, the data for youth aged 4 months–36 years covers almost the full range of development. Thanks to medical data collected from parental groups our research group includes children from all over Poland, not just from one region. The gathered group of over 400 children covers the spectrum of children with many DS-typical diseases.

## Conclusions

There is no single comprehensive tool for the assessment of the developmental disorders in DS. The differences between the results obtained using standard growth charts and specialized ones were identified, however, they are ambiguous in the clinical meaning. It is both type of growth charts that are capable of detecting development disorders early in the broadest possible way. The findings of our study can be valuable for healthcare professionals, parents, and guardians in drawing attention to the need for complex monitoring of developmental disorders in people with DS. Accurate assessment of anthropometric indicators of the development may enable to improve the quality of life and to extend the period of a healthy lifespan.

## Data availability statement

The raw data supporting the conclusions of this article will be made available by the authors, without undue reservation.

## Ethics statement

The studies involving human participants were reviewed and approved by the Bioethics Committee, Wroclaw Medical University (approval number KB 674/2020). Written informed consent to participate in this study was provided by the participants’ legal guardian/next of kin.

## Author contributions

MH: term, Conceptualization, Investigation, Resources, Data Curation, Writing - Original Draft, Project administration, Visualization. HM: Validation, Formal analysis, Data Curation, EB: Methodology, Resources, Writing - Review and Editing, Supervision, Project administration. All authors contributed to the article and approved the submitted version.

## Funding

This research received no external funding. However the publication will be financed by the Wroclaw Medical University, including subsidy funds (Wroclaw Medical University; SUBK.D130.22.055) for the project “Children and young adults with Down Syndrome- metabolomics”.

## Acknowledgments

Our greatest thanks go to the children and their families who participated in this study, the parents’ interest groups and organizations that assisted in reaching those families. We would also like to thank Dr. Chapman and Dr. Madera of King’s College Hospital who were extremely helpful at the very beginning of our research in guiding us to electronic data on standardized growth charts for children with Down’s syndrome. The provided data has been used only for scientific purposes.

## Conflict of interest

The authors declare that the research was conducted in the absence of any commercial or financial relationships that could be construed as a potential conflict of interest.

## Publisher’s note

All claims expressed in this article are solely those of the authors and do not necessarily represent those of their affiliated organizations, or those of the publisher, the editors and the reviewers. Any product that may be evaluated in this article, or claim that may be made by its manufacturer, is not guaranteed or endorsed by the publisher.
